# The Blind Watchmaker

**Published:** 1987-05

**Authors:** Clive Richards


### THE BLIND WATCHMAKER

**Richard Dawkins**
**Longman, London, 1986**
**ISBN 0 582 44694 5. Price £12.95**

One of the advantages of working as a GP is having the opportunity to listen to the car radio during the day. Whilst the journey time between house visits is rarely adequate to include whole musical items, one can often catch the interval reading. I was thus first introduced to Richard Dawkins's new book and became fascinated with his tales from nineteenth century theology interspersed with recent experiments needing superglue to stick false tails on Kenyan widow birds to assess their mating potential!

'The Blind Watchmaker' is about evolution—specifically, the power of processes of cumulative selection to develop biological systems of amazing complexity. It is a tightly argued exposition which sets out to defend the much misunderstood theory of Charles Darwin. It is leavened with a wide variety of examples ranging from echolocation systems in bats to the embryology of the human eye and the replication of DNA. Initial evolutionary changes are illustrated by a series of deceptively simple computer models ('Biomorphs') which the author evolves by random processes in a computer to become different and more complex structures. The whole of the theory of natural selection is covered in small steps written in lucid prose and intelligible even to those of us who thought that Cell Biology and Statistics became extinct species after the Second MB examination.

The most compelling feature of this book is the way that it is written—it is a model of a convincing scientific argument. Dawkins himself states, "Explaining is a difficult art. [This book] seeks to inform and to inspire. I want to inspire the reader with a vision of our own existence as a spine chilling mystery and simultaneously to convey the full excitement of the fact that it is a mystery with an elegant solution that is within our grasp". Certainly this is a fascinating story about Life—before reading it I had not considered the relative running speeds of gazelles and cheetahs to be an 'arms race' between the species with extinction the penalty for losing.

This book is by no means a mainstream medical textbook. Nonetheless, as doctors we all benefit from time to time from looking outwards and seeing mankind with a different perspective and on these grounds alone 'The Blind Watchmaker' can be recommended. Above all it sets high standards in combining explanations from the biological sciences, philosophy and theology with a masterful use of language. Would that many medical books were as well written.

Clive Richards

